# High and Low Adherence to Mediterranean and DASH Diet Patterns and the Risk of Heart Failure: A Meta-Analysis of Observational Studies †

**DOI:** 10.3390/life15010063

**Published:** 2025-01-07

**Authors:** Mehmet Emin Arayici, Mustafa Eray Kilic, Mehmet Birhan Yilmaz

**Affiliations:** 1 Department of Biostatistics and Medical Informatics, Faculty of Medicine, Dokuz Eylül University, 35340 İzmir, Türkiye; 2 Department of Public Health, Faculty of Medicine, Dokuz Eylül University, 35340 İzmir, Türkiye; 3 Department of Cardiology, Faculty of Medicine, Dokuz Eylül University, 35340 İzmir, Türkiye; mustafaeraykilic@gmail.com (M.E.K.); prof.dr.mbyilmaz@gmail.com (M.B.Y.)

**Keywords:** heart failure, Mediterranean diet, DASH diet, meta-analysis

## Abstract

Background. The relationship between heart failure (HF) and Mediterranean and DASH diets is not well delineated. This meta-analysis aimed to assess the effectiveness of high adherence to Mediterranean and DASH diets compared to low adherence in reducing the risk of incident HF (primary prevention of HF) and reducing all-cause mortality in patients with HF (secondary prevention of HF). Methods. The reporting stages of this meta-analysis closely adhered to the PRISMA guidelines. A comprehensive literature search was undertaken for published papers in PubMed, Embase, EBSCO, ICTRP, and the NIH clinical trials databases. Results. A total of 16 reports from 14 studies were included in this paper. A significant inverse association was identified between high adherence to the Mediterranean diet model (compared to low adherence) and the risk of incident HF (OR = 0.77, 95% CI: 0.63–0.93, *p* = 0.007) among patients without previous diagnosis of HF. Similarly, there was a significant and inverse relationship between high adherence to the DASH diet (compared to low adherence) and the risk of incident HF (OR = 0.83, 95% CI: 0.70–0.98, *p* = 0.03) among patients without previous diagnosis of HF. High adherence to the Mediterranean diet model (compared to low adherence) was associated with lower all-cause mortality (OR = 0.88, 95% CI: 0.78–0.99, *p* = 0.03) among patients with HF. Conclusions. This paper demonstrated that high adherence to Mediterranean and DASH diets significantly reduced the risk of incident HF among individuals without a previous diagnosis of HF, whereas only high adherence to the Mediterranean diet was associated with lower all-cause mortality among patients with HF.

## 1. Introduction

It is well-established that heart failure (HF) presents a complex medical challenge, impacting approximately 1–2% of the global adult population [1,2]. The prevalence of HF increases with age and has a significant adverse impact on public health. The prevalence of HF is expected to increase by 46% from 2012 to 2030, elevating the overall HF prevalence from 2.4% to 3.0% [3]. The lifetime risk of HF in the general population varies between 20% and 46% at 45 years of age, with particularly higher rates among individuals with hypertension and a high body mass index (BMI) [4].

According to the World Health Organization (WHO), lifestyle modifications targeted at the prevention and management of risk factors have the potential to reduce approximately 75% of cardiovascular disease (CVD)-related deaths. Unhealthy eating habits, lack of physical activity, smoking, and excessive alcohol consumption were identified as the primary behavioral risk factors associated with cardiovascular disease and stroke [5,6]. Cost-effective interventions are required to improve outcomes in HF. One potential approach may involve nutritional interventions as patients with HF can experience nutritional imbalances and malnutrition, which could contribute to higher morbidity and mortality rates [5]. Previous research including Diet and Reinfarction Trial (DART), Dietary Approaches to Stop Hypertension (DASH), and Prevención con Dieta Mediterránea (PREDIMED) have highlighted the substantial influence of dietary intake on the incidence and severity of CVD [7].

The most beneficial dietary regimen for CVD risk reduction emphasizes the consumption of whole grains, fruits, vegetables, legumes, nuts, fish, poultry, moderate dairy products, and heart-healthy vegetable oils [8]. The Mediterranean diet, which prioritizes plant-based foods and sources of plant protein, stands out as an established favorable dietary pattern for mitigating CVD risk. Meanwhile, the DASH diet, comprising fruits, vegetables, whole grains [9], poultry, fish, nuts, and low-fat dairy items, has gained prominence owing to its capacity to lower blood pressure and potentially prevent left ventricular dysfunction, a common complication associated with hypertension [10,11,12].

The importance of diet in managing HF is increasingly recognized, and the Mediterranean and DASH diets are usable as potential research and intervention strategies for HF in the future [13]. These diets, which are affordable, accessible, and long-term nutritional treatments, may have effects associated with both HF risk and mortality in patients with HF. However, the growing evidence base on the subject has suggested contradictory results for both the Mediterranean diet model [14,15,16] and the DASH diet pattern [17,18,19]. Therefore, the relationship between HF and associated dietary patterns is not clearly understood. The purpose of this review-based study employing systematic and meta-analytic approaches was to comprehensively assess and enlighten the influence of high adherence to Mediterranean and DASH diets compared to low adherence on HF-related outcomes using a meta-analysis method.

## 2. Methods

All reporting stages of this review-based study employing systematic and meta-analytic approaches were carried out in accordance with PRISMA (Preferred Reporting Items for Systematic Reviews and Meta-Analyses) guidelines [20]. The meta-analysis protocol was officially registered with the International Prospective Register of Systematic Reviews (PROSPERO) with the registration number CRD42023427976. The PRISMA checklist has been related to Supplementary Table S1, serving as an essential tool to validate adherence to PRISMA guidelines, ensuring the thoroughness and reporting accuracy of the study.

PICOS framework related to meta-analysis was defined as follows: Population: healthy individuals for HF risk or patients diagnosed with HF for mortality; Intervention: adherence to Mediterranean and DASH diets; Comparison: levels of adherence (high adherence vs. low adherence); Outcomes: reduction in risk of HF, and all-cause mortality in patients with HF; Study Design: cohort or case–control studies.

### 2.1. Information Sources, Search Strategy, and Study Selection Process

Comprehensive literature searches were executed in PubMed/Medline, Embase, EBSCO Academic Search Ultimate, International Clinical Trials Registry Platform (ICTRP), and NIH clinical trials databases. The search strategy was formulated by utilizing Medical Subject Headings (MeSH) and free-text terms that incorporated “HF”, “Mediterranean diet”, “DASH diet”, “hospitalization”, and “mortality”. Boolean operators (AND/OR) were used to combine related keywords. Originally, the search strategy was developed in PubMed and subsequently applied to other databases. Detailed search strategies were documented in Supplemental Table S2. In August 2024, an initial comprehensive literature search was performed, and an update was conducted in October 2024.

### 2.2. Eligibility Criteria

Papers were included if they focused on adults aged 18 years or older who were healthy individuals or who were diagnosed with HF, either systolic and/or diastolic, for this study. To meet the inclusion criteria, the studies had to assess the comparison of adherence to Mediterranean and DASH diets across different quantiles (high and low) and report results for at least one of the following outcomes: HF-related risk or all-cause mortality in patients with HF. Inclusion criteria were limited to studies published in the English language. Papers published in other languages were directly excluded from the study. Studies that met any of the following exclusion criteria were not considered: (i) those with insufficient data or lacking key outcome reporting; (ii) articles categorized as case reports, case series, editorials, comments, or expert opinions; (iii) studies involving animal or in vitro research; and (iv) studies utilizing overlapping or duplicate data sets.

### 2.3. Data Collection Process

Two independent researchers (MEA and MEK) extracted data from the primary papers obtained from relevant databases and recorded it in a predefined Microsoft Excel^®^ spreadsheet. Data extracted covered a variety of parameters, including first author name, publication date, study design, study name, type of diet, sample size, mean or median age, follow-up time, effect size, 95% confidence intervals (CI), *p*-values, and adjusted covariates/confounders. In studies where more than one result was reported, data in the highest quarter/category and the lowest quarter/category were considered, and a multivariate-adjusted model was selected (the latest model with the most factors included). If data were missing or unclear on some topics, we made an effort to contact the corresponding authors of the original articles through email for clarification. To ensure accuracy, both investigators cross-verified all the extracted data and arrived at a consensus.

### 2.4. Quality Assessment in Individual Studies

The methodological quality and risk of bias for the included studies were assessed using standardized tools. For cohort and case–control studies, the Newcastle–Ottawa Scale (NOS) [21] was employed. The NOS scores between 6 and 9 indicated a moderate-to-high quality of the studies involved. Studies with lower scores that were not within this range were excluded from the pooled analysis. To maintain the reliability of this evaluation, both reviewers conducted these assessments independently. If discrepancies arose, discussions were held to reach a consensus, and if required, a third investigator was consulted for mediation.

### 2.5. Statistical Analysis

The statistical analyses were carried out using R software version 4.2.3, employing the “*metafor*” package [22], The Review Manager version 5.4 (The Nordic Cochran Centre, Copenhagen, Denmark) [23], and ProMeta3^®^ meta-analysis software version 3.0 [24]. Effect size (ES) was computed as the odds ratio (OR) for risk of HF and all-cause mortality in patients with HF. The level of heterogeneity among the included studies was quantified using I^2^ statistics or the chi-squared (χ2) test. The I^2^ statistic represents the proportion of variance between studies attributed to heterogeneity rather than random sampling error. Significant heterogeneity was confirmed with a *p*-value of less than 0.05 in the chi-squared (χ2) test and an I^2^ quantitative estimation value exceeding 50%. In the case of low to moderate heterogeneity, fixed effect models were used, whereas random effect models were utilized for high heterogeneity. To assess the potential for bias, Egger’s linear regression test, Begg and Mazumdar’s rank correlation test, as well as funnel plot visualizations, were conducted [25]. Statistical significance was depicted as a two-tailed *p*-value less than 0.05 in all tests performed.

In order to thoroughly investigate the connection between individual dietary components of the Mediterranean and DASH diets and cardiovascular outcomes, with a particular focus on HF risk and all-cause mortality in patients with HF, we conducted a subgroup analysis. To address potential statistical heterogeneity among the included studies, we employed both fixed effects and random effects models with restricted maximum likelihood estimation.

To evaluate the robustness of our results, sensitivity analyses were conducted. This involved reassessing the effect size (ES) by sequentially omitting each study from the pooled analysis, thereby estimating the influence of individual studies on the overall findings.

## 3. Results

### 3.1. Literature Search

**Figure 1:**
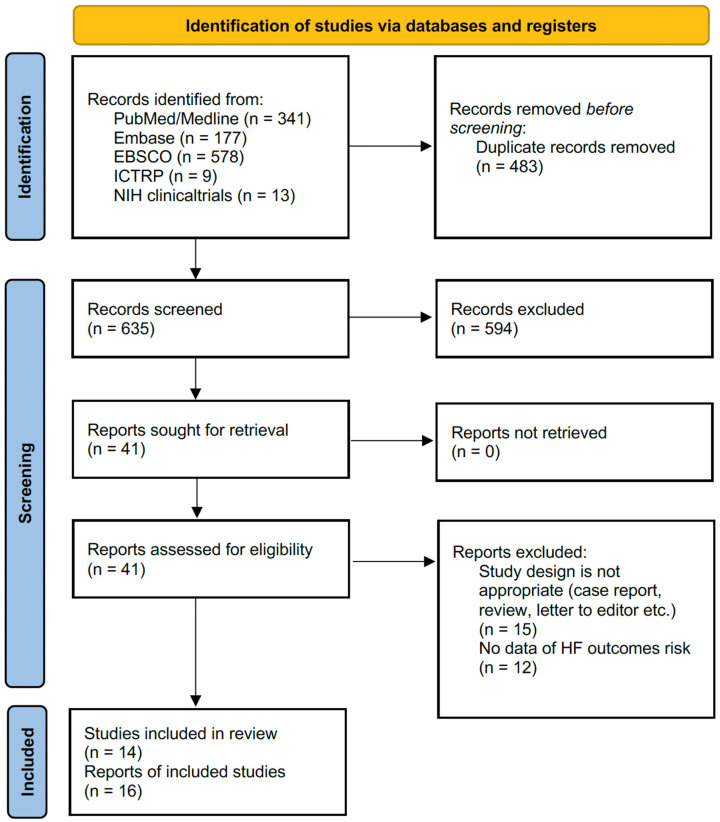
Flow chart for the selection of studies included in the systematic review and the meta-analysis.

The initial search was operated across multiple databases, including PubMed/Medline (*n* = 341), Embase (*n* = 177), EBSCO (*n* = 578), ICTRP (*n* = 9), and NIH clinical trials (*n* = 13), yielding a total of 1118 papers. Out of the total 1118 records identified, 483 of these were found to be duplicates and were subsequently eliminated from consideration. The remaining 635 records underwent a relevance screening process, which involved reviewing the titles and abstracts of each record. Out of these, 41 papers were considered suitable for comprehensive full-text evaluation. Throughout this evaluation, 15 studies were excluded due to unsuitable study designs, and 12 were excluded for the absence of data related to HF mortality or incidence. In the final analysis, a total of 16 reports derived from 14 papers [14,15,16,17,18,19,26,27,28,29,30,31,32,33] met the predefined inclusion criteria and were integrated into the meta-analysis. A flowchart representation of the literature search and study selection process in accordance with PRISMA guidelines is provided in Figure 1.

### 3.2. Baseline Characteristics of Included Studies

This meta-analysis included 16 reports from 14 observational studies [14,15,16,17,18,19,26,27,28,29,30,31,32,33] and a total of 424,502 participants. The studies were conducted in several countries, including Spain, the United States, Sweden, Germany, Italy, and the Netherlands, and they were published between 2014 and 2022. The ages of the participants in all studies were generally middle-aged and older. The majority of studies consisted of population-based and prospective cohort studies. Multiple dietary adherence scores have been utilized across these studies, including the 14-point Mediterranean diet adherence score, the DASH score, the MeDi Score, and the aMed scoring system. Among the studies included were MEDIT-AHF (Mediterranean DieT in Acute HF) [17], Women’s Health Initiative [14], Cohort of Swedish Men [32,33], Swedish Mammography Cohort [16,18], EPIC (European Prospective Investigation into Cancer and Nutrition) [15,31], REGARDS (REasons for Geographic and Racial Differences in Stroke) [19], MESA (The Multi-Ethnic Study of Atherosclerosis) [29], Cardiovascular Health Study [26], SCCS (Southern Community Cohort Study) [30], and NHANES (National Health and Nutrition Examination Survey) [27,28].

The follow-up periods in the included studies ranged from 2.1 years in the MEDIT-AHF study to 21.5 years in the Cardiovascular Health Study, with an overall average of 8.9 years. The two main dietary therapies studied in these trials were the Mediterranean and DASH diets. Diet adherence was frequently assessed using a variety of scoring techniques, with people with poorer adherence scores serving as comparator groups. In general, in the DASH diet scoring, food groups including fruits, vegetables, whole grains, nuts, legumes, and low-fat dairy products are scored positively. Red and processed meats, sweetened beverages, and sodium were listed as negative food groups and reverse scored [14,18,26,29,33]. Yielding considerable clinical heterogeneity was emerging between studies regarding dietary scoring. In some studies, dietary adherence was divided into four groups or five quantiles [14,18,29,33], while in another study, diet compliance was divided into two groups [27]. In studies related to the Mediterranean diet pattern, participants consumed moderate amounts of alcohol (10–25 g/day for men and 5–15 g/day for women), seafood, whole grains, legumes, monounsaturated fat + polyunsaturated fat/saturated fat ratio, nuts, fruits, and vegetables were determined as positive foods. Consumption of red and processed meat, dairy, and more than moderate amounts of alcohol were scored negatively. Diet adherence was assessed utilizing different scoring scales in some studies, divided into two groups [17,18], and in some studies, divided into three or four categories [15,16,31]. These studies scrutinized outcomes such as HF-related hospitalizations, HF risk, and all-cause deaths or cardiovascular deaths. The baseline characteristics and study designs of the included investigations are associated with Table 1.

The adherence to DASH and Mediterranean diets has been assessed using diverse methodological approaches across the included studies, contributing to clinical heterogeneity in evaluating dietary impacts. Self-administered FFQs were frequently employed, as seen in Levitan et al. [14,18,33] and Del Gobbo et al. [26], focusing on DASH components like fruits, vegetables, and sodium intake. Modified block FFQs, used in the Women’s Health Initiative studies [14], included both DASH and Mediterranean dietary patterns, adapting for regional food availability. For the Mediterranean diet, adherence was assessed using tools like the PREDIMED questionnaire, modified Mediterranean Diet Score (mMED), and traditional Mediterranean Diet Score (MeDi) [16,17,34]. Semi-quantitative FFQs were common in Mediterranean diet assessments, as applied in studies like Wirth et al. [15] and Tektonidis et al. [16,32], highlighting core components such as olive oil, fish, and nuts. The PREDIMED questionnaire, a specialized tool for Mediterranean dietary patterns, was used by Miro et al. (2018) [17], emphasizing the diet’s unique elements like extra virgin olive oil and nuts. Other studies, such as Chang et al. (2022) [28] and Chou et al. (2022) [27], relied on 24 h dietary recall interviews to provide detailed dietary intake data, albeit with increased reliance on participant memory. Campos et al. [29] and Goyal et al. [19] employed extended FFQs, offering comprehensive dietary analysis.

The NOS scores for the included 14 observational studies varied between 6 and 9, indicating a moderate-to-high quality of the studies involved. A detailed quality assessment is summarized in Supplementary Table S3.

### 3.3. Results of the Meta-Analysis

A meta-analysis was executed that yielded data from eight reports on the Mediterranean diet and eight studies on the DASH diet. Among the Mediterranean diet, five reports from four studies focused on evaluating the risk of HF [15,16,31,32], while three studies focused on assessing all-cause mortality in patients with HF [14,17,28]. In the DASH diet, six studies reported results related to the risk of HF [18,19,26,29,30,33], while two studies reported results related to mortality in patients with HF [14,27].

### 3.4. Outcomes of the Meta-Analysis on Incident Heart Failure Risk

**Figure 2:**
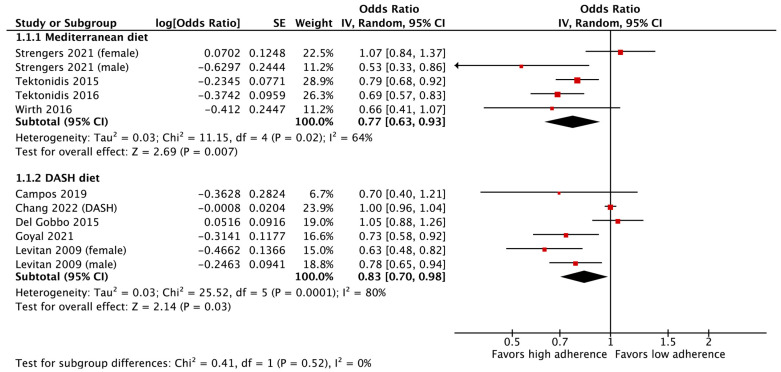
The forest plot of the effect of the Mediterranean diet and the DASH diet pattern on the risk of heart failure [15,16,18,19,26,29,30,31,32,33].

In the pooled meta-analysis utilizing a random effects model, a significant inverse association was identified between high adherence to the Mediterranean diet model (compared to low adherence) and the risk of incident HF among patients without a previous diagnosis of HF (OR = 0.77, 95% CI: 0.63–0.93, *p* = 0.007) (Figure 2). Moderate and significant heterogeneity was observed in the studies assessing the Mediterranean diet and the risk of incident HF (Tau^2^ = 0.03, Chi^2^ = 11.15, I^2^ = 64%, *p* = 0.02). The analyses conducted showed no significant evidence of bias. This outcome was confirmed by the results of Egger’s test (Intercept = −1.01, t = −0.50, *p* = 0.65) and Begg and Mazumdar’s rank correlation test (z = −0.49, *p* = 0.62). The visualization of the funnel plot is illustrated in Supplemental Figure S1.

Similarly, in the pooled analysis employing a random effects model, it was revealed that there was a significant and inverse association between high adherence to the DASH diet pattern (compared to low adherence) and the risk of incident HF among patients without a previous diagnosis of HF (OR = 0.83, 95% CI: 0.70–0.98, *p* = 0.03) (Figure 2). Significant and substantial heterogeneity was detected within the studies evaluating the DASH diet in relation to the risk of incident HF risk (Tau^2^ = 0.03, Chi^2^ = 25.52, I^2^ = 80%, *p* < 0.001). Hence, in light of the noticeable heterogeneity, the analysis was conducted utilizing the random effects model. Analyses indicated no evidence of bias, as confirmed by Egger’s test (Intercept = −2.28, t = −2.60, *p* = 0.06) and Begg and Mazumdar’s rank correlation test (z = −1.69, *p* = 0.09). The visualization of the funnel plot is provided in Supplemental Figure S1.

### 3.5. Outcomes of the Meta-Analysis on Mortality in Patients with Heart Failure

**Figure 3:**
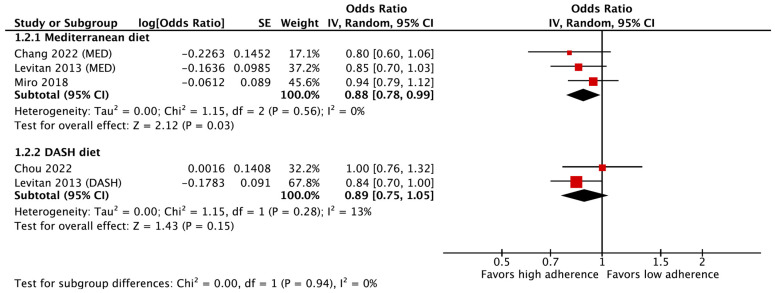
The forest plot of the effect of the Mediterranean diet and the DASH diet pattern on all-cause mortality in patients with heart failure [14,17,27,28].

In the meta-analysis, which employed a fixed effects model, a noteworthy inverse association was observed between high adherence to the Mediterranean diet model (compared to low adherence) and all-cause mortality among patients with HF (OR = 0.88, 95% CI: 0.78–0.99, *p* = 0.03) (Figure 3). No significant heterogeneity was evident in the studies examining the relationship between the Mediterranean diet and all-cause mortality in patients with HF (Tau^2^ = 0.00, Chi^2^ = 1.15, I^2^ = 0.0%, *p* = 0.56). Furthermore, the analyses carried out did not reveal any substantial evidence of bias. This conclusion was supported by the results of Egger’s test (Intercept = −2.62, t = −1.49, *p* = 0.37) and Begg and Mazumdar’s rank correlation test (z = −1.57, *p* = 0.11). The visual examination of the funnel plot is presented in Supplemental Figure S2).

In a pooled analysis of two independent studies examining high adherence to the DASH diet model (compared to low adherence) and all-cause mortality in patients with HF, no significant association was found between the DASH diet and all-cause mortality among patients with HF (OR = 0.89, 95% CI: 0.75–1.05, *p* = 0.15) (Figure 3). It appears that no significant and substantial heterogeneity was observed within the studies assessing the DASH diet in relation to the all-cause mortality in patients with HF (Tau^2^ = 0.00, Chi^2^ = 1.15, I^2^ = 13%, *p* = 0.28). Since there were only two reports, bias analysis was not conducted.

### 3.6. Subgroup Analysis

We executed various subgroup analyses from eligible studies to address individual dietary components of the Mediterranean and DASH diet patterns in terms of incident HF risk among patients without previous diagnosis of HF and all-cause mortality among patients with HF. Consistently across all the analyzed data, it was observed that a high-quantile consumption of fruits and legumes was linked to a notably reduced risk of incident HF (Table 2). Moderate alcohol intake, particularly wine—a characteristic feature of the Mediterranean diet—also exhibited a protective effect against incident HF risk. Of note, consumption of legumes and fruits was linked to decreased incident HF risk as well. On the contrary, a high-quantile consumption of vegetables and fish did not exhibit a statistically significant association with incident HF risk. However, consumption of vegetables and less dairy consumption were significantly associated with reduced all-cause mortality among patients with HF (Table 3). Of note, low sodium did not have any significant impact on all-cause mortality risk among patients with HF.

### 3.7. Sensitivity Analysis

In the sensitivity analyses designed to test the stability of our findings, we employed a sequential exclusion method where each study was individually removed from the pooled analysis to observe the effect on the effect size (ES). The subsequent re-evaluation of the ES after each study’s exclusion revealed minimal fluctuations, thereby affirming the robustness of our initial findings. The outcomes of these analyses are documented in Supplemental Figures S3 and S4. The outcome suggests that our results are not disproportionately affected by any single study included in the meta-analysis.

## 4. Discussion

The aim of this meta-analysis was to clarify the impact of high adherence to the Mediterranean and DASH diets on the incident HF risk and all-cause mortality among individuals with HF. A substantial body of data consisting of 16 reports of 14 studies with a total sample size of 424,502 participants was examined, confirming a beneficial connection between adherence to Mediterranean and DASH dietary patterns and a decreased risk of incident HF among individuals without a previous diagnosis of HF. Notably, high adherence to a Mediterranean diet was furthermore associated with a lower rate of overall mortality in patients with HF. However, there was insufficient evidence regarding the influence of the DASH diet on all-cause mortality among patients with HF.

According to the DASH diet [29,30], people should eat less red meat, fat, and sugar and more fruits, vegetables, grains, grain products, lean meats, fish, and poultry, low- or nonfat dairy products, nuts, seeds, and legumes. They should also limit their intake of sodium. This diet was first recommended for hypertension, though, our meta-analysis has indicated on top of the existing literature that the DASH diet prevents incident HF by 17%. The DASH diet may help prevent HF by lowering blood pressure and preventing coronary heart disease.

The molecular mechanisms underlying the protective effects of the Mediterranean and DASH diets involve complex interactions between dietary components and key cellular pathways. The Mediterranean diet, through its high content of polyphenols (e.g., oleuropein, hydroxytyrosol), modulates epigenetic regulators such as DNA methyltransferases and histone deacetylases, leading to alterations in gene expression that suppress inflammatory and oxidative stress responses [35]. These compounds also inhibit nuclear factor-κB (NF-κB) activation, a key driver of pro-inflammatory cytokine production, thereby attenuating chronic low-grade inflammation implicated in cardiovascular diseases and neurodegenerative disorders [36]. Furthermore, olive oil, a cornerstone of the Mediterranean diet, contains phenolic compounds like hydroxytyrosol, which inhibit inflammatory enzymes such as COX-2 and MMP-9 while suppressing PKCα and PKCβ1 pathways in monocytes, thereby offering vascular protection [36]. Similarly, the DASH diet’s emphasis on potassium, magnesium, and bioactive antioxidants from fruits and vegetables reduces oxidative damage by neutralizing free radicals and enhancing glutathione synthesis, critical for maintaining cellular redox homeostasis [37]. Additionally, both diets influence metabolic pathways linked to lipid metabolism and vascular health. The Mediterranean diet’s monounsaturated fatty acids (MUFAs) from olive oil and omega-3 fatty acids from fish suppress lipogenesis and improve endothelial function by enhancing nitric oxide bioavailability while reducing adhesion molecule expression, which mitigates atherosclerotic plaque development [38]. The DASH diet’s low sodium content and high potassium-to-sodium ratio further contribute to blood pressure reduction by modulating renal sodium handling and reducing vascular resistance [37]. Both dietary patterns underscore the pivotal role of nutritional components in modulating inflammatory and oxidative pathways, thereby fostering systemic health and preventing chronic disease progression [38]. Collectively, these molecular and cellular effects highlight the synergistic action of dietary components in preventing metabolic and inflammatory diseases while promoting longevity and optimal health.

The protective effects of the Mediterranean and DASH diets on cardiovascular health are not merely confined to their well-known antihypertensive benefits. Both dietary patterns also impart additional cardiovascular advantages, such as enhancing diastolic function and ameliorating arterial stiffness [28,39]. Importantly, these diets could mitigate oxidative stress—a key antecedent to HF. The composition of these diets, abundant in fruits, vegetables, whole grains, and lean proteins, lends itself to these cardioprotective effects [29]. However, the inclusion of dairy products in the DASH diet remains a subject of ongoing scrutiny, as its role in HF is yet to be understood [30].

The Mediterranean diet is particularly lauded for its anti-inflammatory and antioxidant properties, contributing to its inverse relation with HF severity. Mechanistically, this diet may exert its beneficial effects through the suppression of proinflammatory markers like IL1β, IL1RN, TNF-α, ICAM1, hs-CRP, and IL-6 [16,31,34]. Furthermore, it is worth noting that certain nutrients within the Mediterranean diet, such as mono-unsaturated fatty acids, may have the capacity to inhibit detrimental metabolic shifts in cardiac function, thereby mitigating HF risk [33]. Both diets are also commendable for their low sodium content, a critical element in preventing incident HF. Moreover, there is emerging evidence to suggest that these diets, replete with antioxidants and micronutrients, could influence the gut microbiome in a manner that offers additional protection against HF [40].

Hence, both the Mediterranean and DASH diets appear to offer broad-spectrum cardiovascular benefits that extend beyond their well-established antihypertensive effects. These findings accentuate the utility of these dietary patterns in both the prevention and management of HF and beckon further rigorous research to validate their roles comprehensively. Of note, the Mediterranean diet appears to positively influence sleep-disordered breathing (SDB) and its downstream effects on diastolic function, mechanisms that are particularly relevant to HFpEF. Studies have demonstrated that adherence to the Mediterranean diet, particularly when combined with physical activity, significantly reduces the apnea–hypopnea index during REM sleep in patients with obstructive sleep apnea syndrome (OSAS). This improvement is largely attributed to reductions in central obesity and metabolic risk markers, which are key contributors to SDB pathophysiology [41]. Moreover, systemic inflammation, which is prevalent in SDB and HFpEF patients, has been directly associated with diastolic dysfunction. The Mediterranean diet’s anti-inflammatory properties may mitigate these effects, improving diastolic performance in patients with both conditions [42]. Additionally, weight loss interventions based on the Mediterranean diet have shown significant reductions in oxidative stress and inflammatory markers when combined with CPAP therapy, suggesting a synergistic effect in alleviating SDB-related cardiovascular dysfunction [43]. Furthermore, the Mediterranean diet’s ability to enhance endothelial function and reduce diastolic blood pressure may contribute to its cardioprotective effects, particularly in improving diastolic function in patients with HFpEF [44]. These findings highlight the potential of the Mediterranean diet as a non-pharmacological intervention to address the interplay between SDB, inflammation, and diastolic dysfunction, offering a promising strategy for managing HFpEF and related comorbidities.

The Mediterranean diet plays a significant role in managing obesity, a critical contributing factor to arrhythmia-induced HF, and its preventive and therapeutic potential is increasingly recognized [45,46]. Obesity is a well-established risk factor for atrial fibrillation, the most common arrhythmia leading to HF, due to its effects on left atrial enlargement, systemic inflammation, and oxidative stress [45]. The Mediterranean diet, characterized by high consumption of fruits, vegetables, whole grains, nuts, olive oil, and lean proteins, particularly fish, has demonstrated efficacy in addressing obesity through caloric balance, improved satiety, and metabolic regulation. Research indicates that adherence to the Mediterranean diet reduces body weight, waist circumference, and visceral adiposity, factors directly linked to atrial remodeling and arrhythmogenesis. For instance, weight loss facilitated by the Mediterranean diet is associated with reduced left atrial volume and improved atrial conduction, lowering AF risk and its progression to HF [41]. Furthermore, the diet’s anti-inflammatory and antioxidant properties mitigate the systemic inflammatory burden and oxidative damage that exacerbate arrhythmogenesis and cardiac remodeling [43]. The Mediterranean diet also improves lipid profiles and glycemic control, reducing other obesity-related risk factors such as hypertension and diabetes, which further contribute to arrhythmia-induced cardiac dysfunction. By addressing both obesity and its downstream effects, the Mediterranean diet emerges as a holistic strategy for preventing and managing arrhythmia-induced HF, supporting its integration into comprehensive cardiac care. Future research should explore its role in specific patient subgroups to optimize its therapeutic potential further.

Epicardial adipose tissue (EAT) is a metabolically active fat depot closely associated with myocardial function, and its inflammation and expansion are critical contributors to cardiac dysfunction. The anti-inflammatory properties of the Mediterranean diet, driven by its high content of omega-3 fatty acids, polyphenols, and monounsaturated fats, reduce the pro-inflammatory cytokine profile of EAT, including TNF-α and IL-6, which are known to promote myocardial fibrosis and dysfunction [47]. Furthermore, the diet improves insulin sensitivity and lipid metabolism, addressing the impaired glucose and lipid handling observed in EAT of heart failure patients, which exacerbates cardiac stress [48]. Reductions in EAT volume associated with adherence to the Mediterranean diet decrease mechanical stress and inflammatory signaling in the myocardium, contributing to improved cardiac function and reduced risk of heart failure [49]. Additionally, the Mediterranean diet enhances the secretion of protective adipokines, such as adiponectin, which counteracts oxidative stress and inflammation, supporting myocardial health [50]. These findings underscore the therapeutic potential of the Mediterranean diet in targeting EAT as a modifiable risk factor, providing a promising avenue for managing heart failure pathophysiology, particularly in conditions like HFpEF. Therefore, it is suggested that this type of plant-based diet contributes significantly to its potential effect on HF and is consistent with our outcomes.

Of note, resveratrol, a polyphenolic compound found in grapes and red wine, exhibits cardioprotective properties that may hold significant therapeutic potential in the management of HF. Resveratrol’s benefits stem from its ability to modulate multiple molecular pathways involved in oxidative stress, inflammation, and cardiac remodeling. Research has demonstrated that resveratrol enhances nitric oxide bioavailability and upregulates proteins such as endothelial nitric oxide synthase and inducible nitric oxide synthase, which contribute to improved myocardial function and reduced ischemic damage [51]. Additionally, resveratrol acts as a potent antioxidant, attenuating oxidative stress and inflammation through pathways like PI3K/Akt and AMPK, while also promoting autophagy to remove damaged cellular components [52]. In experimental models of HF, resveratrol has been shown to improve left ventricular function, reduce fibrosis, and prevent pathological cardiac hypertrophy by regulating stress signaling pathways and oxidative markers such as COX-2 and ROS [53]. Its ability to modulate inflammasome activation and mitochondrial function further underscores its protective role against myocardial injury and arrhythmias, as evidenced by reductions in atrial fibrillation susceptibility in HF models [54]. Collectively, these findings suggest that resveratrol’s multifaceted cardioprotective mechanisms make it a promising candidate for dietary or pharmacological intervention in HF, particularly when integrated into broader lifestyle modifications. Further clinical studies are warranted to validate these effects and establish optimal dose strategies. Taken together, plant-based dietary patterns contain abundant resveratrol; thus, their potential benefits in heart failure are compatible with our study. It sheds light on the long-term effects in HF.

The high heterogeneity observed in analyses of the DASH diet warrants deeper exploration to identify potential sources of variability. Population differences, such as genetic predispositions, baseline dietary habits, and cultural food practices, likely contribute significantly to this heterogeneity. For example, studies show that the impact of the DASH diet on cardiometabolic markers varies across populations due to differences in baseline health status and dietary environments [55]. Furthermore, varying definitions and measures of dietary adherence, such as the use of self-reported food frequency questionnaires versus biomarkers, introduce inconsistencies that complicate the aggregation of findings [56]. Reliance on observational studies introduces potential confounding factors that may bias the results. Unmeasured lifestyle variables, such as physical activity levels, alcohol consumption, and smoking, or socioeconomic factors like education and income, could independently influence the observed benefits of the DASH diet. For instance, higher adherence to the DASH diet is often associated with greater health awareness and access to healthcare, which may independently reduce disease risk [57]. Addressing these confounders through stratified analyses or the inclusion of comprehensive demographic and lifestyle data can help improve the validity of future studies. By standardizing adherence metrics and employing diverse, representative samples in future randomized controlled trials, researchers can reduce heterogeneity and strengthen the evidence base. Moreover, integrating qualitative assessments to understand barriers to adherence can provide actionable insights for tailoring dietary interventions across different populations.

The limited representation of low- and middle-income countries (LMICs) in dietary intervention studies, including those examining the DASH and Mediterranean diets, presents a notable limitation. Dietary patterns, food availability, and socioeconomic factors in LMICs differ significantly from those in high-income countries, which could influence the generalizability of findings. For instance, the affordability and accessibility of foods emphasized in these diets, such as fresh produce and lean proteins, may pose significant challenges in LMICs, potentially reducing adherence and effectiveness [57]. Future research should prioritize studies in these settings to understand how socioeconomic and cultural factors modify the impact of such diets and explore locally appropriate adaptations. Moreover, variability in dietary adherence scoring methods across studies introduces clinical heterogeneity that complicates the synthesis of results. Adherence is often assessed using tools such as food frequency questionnaires or biomarkers, each with distinct limitations, including recall bias or variations in cutoff thresholds for adherence [56]. Standardizing scoring methods and employing more objective measures of adherence, such as food diaries or nutrient biomarkers, would improve the comparability and reliability of findings across diverse populations and study designs. Addressing these limitations will enhance the global applicability and clinical relevance of dietary intervention research.

This meta-analysis examining high and low adherence to the Mediterranean and DASH diets has several limitations that need to be addressed. One of the important limitations of the study is the use of cohort studies, which likely result in the relationship between diet and outcomes being affected by confounding factors. All studies are observational, and observational studies on diet are very prone to bias and confounders. Other limitations include significant disparities in the comparison groups, different methodologies employed to evaluate adherence, inherent biases in food intervention trials, and the majority of studies conducted in high-income countries. As a result, the generalizability of the findings to low- and middle-income countries, where dietary patterns and food options may significantly differ, may be affected. Especially, given the diversity in dietary assessment tools and methodologies used in nutritional research, it is reasonable to suppose that there might not be uniformity across studies in defining levels of adherence. This potential inconsistency can introduce significant clinical heterogeneity, complicating the interpretation of results and the comparison of outcomes across different investigations. This heterogeneity makes it challenging to draw firm conclusions about the effectiveness of these dietary patterns in preventing or managing specific health conditions, such as cardiovascular disease and HF. To address this issue, future research should aim for greater standardization in the assessment of diet adherence. The study also presents inconsistencies in findings regarding the relationship between alcohol intake and cardiovascular disease. These inconsistencies are attributed to the lack of comprehensive evaluation and inconsistent data reporting among the analyzed studies. The utilization of a simplistic binary scale to assess alcohol intake and the absence of gender-based adjustments for variables such as BMI and physical activity further complicate the interpretation of the results.

Another notable limitation is the scarcity of studies reporting on all-cause mortality outcomes for Mediterranean and DASH diets. Only three studies investigated the impact of the Mediterranean diet on all-cause mortality in HF patients, while only two studies examined the DASH diet. This lack of studies addressing this crucial clinical outcome may undermine the statistical power and precision of our estimates. Despite these limitations, the sensitivity analysis demonstrates the robustness of the overall results. No individual study exerted an abnormal influence on the outcomes, thereby lending credibility to the findings. However, these limitations underscore the necessity for more uniform and standardized reporting in future dietary intervention studies, particularly in terms of defining and quantifying dietary adherence. The inclusion of more studies in various contexts is imperative to enhance the generalizability of the findings.

## 5. Conclusions

The findings of this meta-analysis suggest that high adherence to the Mediterranean and DASH diets is associated with a considerable reduction in the incidence of HF compared to low adherence. High adherence to the Mediterranean diet has also been related to decreased all-cause mortality among patients with HF compared to low adherence. However, high DASH diet adherence did not yield a significant reduction in all-cause mortality among patients with HF. Of note, adherence to some components of both diets was more closely related to the lower risk, such as consumption of fruits, legumes, and moderate alcohol, which was linked to decreased incident HF, whereas only consumption of vegetables and less dairy persisted in decreasing mortality risk as a secondary prevention in patients with HF.

## Figures and Tables

**Table 1:** Baseline characteristics of studies included in the systematic review and meta-analysis.

First Author/Year	Study Type	Study Name	Sample Size (*n*)	Age Range (Years)	Events (*n*)	Event of Death (*n*)	Questionnaire	Type of Diet	Follow-Up Time (Years)	Outcome
Levitan et al., 2009 [33]	Cohort	Cohort of Swedish Men	38,987	45–79	710	97	Self-administered FFQ	DASH	9	Incidence
Levitan et al., 2009 [18]	Cohort	Swedish Mammography Cohort	36,019	48–83	415	28	Self-administered FFQ	DASH	7	Incidence
Levitan et al., 2013 [14]	Cohort	Women’s Health Initiative	161,808	50–79	3215	1385	Modified block FFQ	MED	4.6	All-cause mortality
Levitan et al., 2013 [14]	Cohort	Women’s Health Initiative	161,808	50–79	3215	1385	Modified block FFQ	DASH	4.6	All-cause mortality
Del Gobbo et al., 2015 [26]	Cohort	Cardiovascular Health Study	5201	≥65	1380	N/A	99-item FFQ	DASH	21.5	Incidence
Tektonidis et al., 2015 [16]	Population based cohort	Swedish Mammography Cohort	32,921	48–83	1648	N/A	FFQ	MED	10.4	Incidence
Wirth et al., 2016 [15]	Prospective population-based cohort	EPIC	24,008	35–65	209	N/A	Semi-quantitative, self-administered FFQ	MED	8.2	Incidence
Tektonidis et al., 2016 [32]	Population-based cohort	Cohort of Swedish Men	37,308	45–79	1269	146	96-item semi-quantitative, self-administered FFQ	MED	10.9	Incidence
Miro et al., 2018 [17]	Prospective cohort study	MEDIT-AHF	991	N/A	N/A	569	PREDIMED questionnaire	MED	2.1	All-cause mortality
Campos et al., 2019 [29]	Cohort	MESA	4478	45–84	179	N/A	120-item FFQ	DASH	13	Incidence
Strengers et al., 2021 (a) [31]	Cohort	EPIC-NL	9316	21–64	144	N/A	Semi-quantitative FFQ	MED	15	Incidence
Strengers et al., 2021 (b) [31]	Cohort	EPIC-NL	27,645	40–70	489	N/A	Semi-quantitative FFQ	MED	15	Incidence
Goyal et al., 2021 [19]	Cohort	REGARDS	18,856	≥45	767	111	FFQ	DASH	10.1	Incidence
Chang et al., 2022 [28]	Population-based cohort	NHANES	832	≥18	832	319	24 h dietary recall interview	MED	4.7	All-cause mortality
Chang et al., 2022 [30]	Prospective cohort study	SCCS	25,300	40–79	7045	N/A	89 food items 24 h dietary recall questionnaires	DASH	11	Incidence
Chou et al., 2022 [27]	Population-based cohort	NHANES	832	≥18	832	319	24 h dietary recall interview	DASH	4.7	All-cause mortality

DASH The Dietary Approaches to Stop Hypertension, N/A not available, FFQ food frequency questionnaire, EPIC European Prospective Investigation into Cancer and Nutrition, MED Mediterranean diet, MEDIT-AHF Mediterranean DieT in Acute Heart Failure, PREDIMED Prevención con Dieta Mediterránea, MESA Multi-Ethnic Study of Atherosclerosis, NL Netherlands, (a) male, (b) female, REGARDS REasons for Geographic And Racial Differences in Stroke, NHANES National Health and Nutrition Examination Survey, SCCS Southern Community Cohort Study.

**Table 2:** Subgroup analyses of dietary components and heart failure risk of eligible studies included in the systematic review and meta-analysis.

Analysis	Analysis Model	Number of Reports (*n*)	Effect Size (OR)	95% CI	*p* Value	I^2^	*p* Value
Fruits	Fixed	4	0.92	0.85–0.99	0.03	0.00%	0.76
Legumes	Fixed	4	0.93	0.86–0.99	0.04	0.01%	0.48
Moderate Alcohol	Fixed	4	0.91	0.83–0.98	0.02	0.03%	0.38
Vegetables	Fixed	4	0.97	0.85–1.08	0.61	60.48%	0.07
Fish	Fixed	4	0.94	0.86–1.01	0.11	0.00%	0.54
Less Dairy	Fixed	3	0.92	0.84–1.00	0.05	0.08%	0.19
Fiber	Fixed	2	0.92	0.84–1.00	0.06	0.00%	0.89

CI confidence interval, OR odds ratio.

**Table 3:** Subgroup analyses of dietary components and all-cause mortality of eligible studies included in the systematic review and meta-analysis.

Analysis	Model	Number of Reports (*n*)	Effect Size (RR)	95% CI	*p* Value	I^2^	*p* Value
Fruits	Fixed	3	0.99	0.90–1.09	0.99	0.77%	0.36
Legumes	Fixed	3	0.89	0.78–1.00	0.06	24.78%	0.27
Moderate Alcohol	Random	2	1.07	0.78–1.36	0.62	75.28%	0.04
Vegetables	Fixed	3	0.82	0.72–0.92	0.001	0.00%	0.56
Fish	Fixed	2	1.01	0.86–1.16	0.87	0.00%	0.69
Less Diary	Fixed	2	0.80	0.65–0.95	0.01	0.00%	0.78
Less sodium	Fixed	2	1.09	0.94–1.25	0.25	0.00%	0.77

CI confidence interval, RR risk ratio.

## Data Availability

The datasets used and/or analyzed in this study are available upon reasonable request from the corresponding author.

## Supplementary Materials

The following supporting information can be downloaded at: https://www.mdpi.com/article/10.3390/life15010063/s1, Table S1. The PRISMA checklist. Table S2. Search strategy used for the systematic review in PubMed, Embase, EBSCO Academic Search Ultimate, ICTRP, and NIH clinical trials databases. Table S3. Quality assessment of selected observational studies included in meta-analysis (Newcastle–Ottawa Scale). Figure S1. The funnel plot of the effect of the Mediterranean diet and the DASH diet on the risk of heart failure is approximately symmetrical and, in accordance with the results of Egger’s (*p* = 0.65) fades the possibility of potential publication bias. Figure S2. The funnel plot of the effect of the Mediterranean diet and the DASH diet on mortality is approximately symmetrical and, in accordance with the results of Egger’s (*p* = 0.06) fades the possibility of potential publication bias. Figure S3. Sensitivity analysis of the effect of the Mediterranean diet on the risk of heart failure. Figure S4. Sensitivity analysis of the effect of the DASH diet on the risk of heart failure.
